# Caractéristiques des accidents du travail et devenir des victimes: à propos de 133 cas déclarés auprès de la Caisse de Sécurité Sociale de Dakar (Sénégal)

**DOI:** 10.11604/pamj.2018.30.156.10517

**Published:** 2018-06-21

**Authors:** Sidy Akhmed Dia, Azhar Salim Mohamed, Fatou Sy Gaye, El Hadj Oumar Ndoye, Mame Coumba Gaye Fall, Mouhamed Nanibolio Soumah, Mor Ndiaye

**Affiliations:** 1 Service de Médecine Légale et de Travail, Faculté de Médecine, de Pharmacie et d’Odontologie, Université Cheikh Anta Diop de Dakar, Dakar Fann, Sénégal; 2 Hopital Général de Grand Yoff, Dakar, Sénégal

**Keywords:** Accident du travail, indemnisation, invalidité, conséquences, famille, Sénégal, Work-related injuries, compensation, disability, consequences, family, Senegal

## Abstract

**Introduction:**

Les accidents du travail (AT) ont des répercussions sur le système de sécurité social du pays, sur les victimes elles-mêmes ainsi que leurs proches. L’objectif de notre étude est de décrire les différentes caractéristiques des AT déclarés auprès de la Caisse de Sécurité Sociale (CSS) de Dakar et le devenir socio-professionnel des victimes.

**Méthodes:**

Etude rétrospective et transversale sur une période de 5 ans, de 2002 à 2006. Ont été colligés 133 cas sur 9.308 AT déclarés à la CSS. Divers paramètres ont été étudiés: caractéristiques socio-professionnelles des victimes et aspects lésionnels des accidents. Les victimes ont été interrogées pour recueillir leur vécu socio-professionnel, la situation professionnelle.

**Résultats:**

L’âge moyen des sujets était de 37,55 ans avec une prédominance masculine (99,22%). Les secteurs du BTP (22,6%) et de la pêche (15%) étaient les plus touchés. Près des deux tiers des accidents survenaient au lieu du travail (77,4%). Ces accidents entrainaient des fractures dans 51,7% des cas et touchaient la main dans 30,1% des cas. Près de deux tiers des accidentés (60,9%) avaient repris la même profession. On notait 31 cas de licenciement et 12 cas de reclassement professionnel. La durée d’incapacité temporaire totale moyenne était de 236,7 jours. Le montant annuel moyen de la rente était de 1.640.329 Francs CFA (2 504,31 Euros).

**Conclusion:**

Les AT au Sénégal ne présentent pas de grandes particularités comme décrites partout ailleurs. Les faibles ressources allouées aux victimes et l’absence de politique d’accompagnement social expliquent les différentes souffrances des victimes et leurs familles.

## Abstract (en)

**Introduction:**

Work-related injuries have an impact on social security system of the country, on the victims as well as on their relatives. The objective is the aim of our study was to describe the different characteristics of work-related injuries reported to the Social Security Fund (CSS) in Dakar as well as the socioprofessional course of the victims.

**Methods:**

We conducted a retrospective cross-sectional study over a period of 5 years, from 2002 to 2006. Data were collected from 133 cases out of 9 308 reported to the CSS. Various parameters were studied: the socioprofessional characteristics of victims and the lesional features of the injuries. The victims were interviewed to gather informations about their socioprofessional history and their professional situation.

**Results:**

The average age of patients was 37,55 years, with a male predominance (99,22 %). BPW (22.6%) and fishing (15%) sectors were most affected. Almost two thirds of injuries happened in the workplace (77.4%). These injuries resulted in fractures in 51.7% of cases and affected the hand in 30.1% of cases. Nearly two thirds of injured subjects (60.9%) had resumed their profession. Thirty one subjects were fired and 12 were redeployed. The average period of temporary total incapacity was 236.7 days. The median annual pension was 1 640 329 CFA Francs (2 504,31 Euros).

**Conclusion:**

In Senegal, work-related injuries don’t have specific characteristics, as described elsewhere. The low resources allocated to the victims and the lack of supporting social-policy measures explain the different types of suffering of the victims and their families.

## Introduction

L’Organisation Mondiale de la Santé (OMS) définit les accidents du travail (AT) comme étant un problème de santé publique dans les pays en développement [1, 2]. Les souffrances humaines dues à ces accidents ont des dommages chez l’employé, l’employeur et la société [3, 4]. Selon le Bureau International du Travail (BIT), il se produit chaque année dans le monde près de 268 millions AT par an [5-8]. Pour les victimes, les AT sont responsables de: douleurs, souffrance endurée, handicap, réadaptation, perte de performance à la reprise, perte de salaire ou perte d’emploi. Des répercussions sont parfois notées au niveau des familles des victimes: diminution du pouvoir d’achat, conflits, dislocation du tissu familial… [9, 10]. Au Sénégal, l’AT est définit comme étant tout évènement soudain, quelle qu’en soit la cause, l’accident survenue à un travailleur: par le fait ou à l’occasion du travail; pendant le trajet de sa résidence au lieu de travail et vice-versa, dans la mesure où le parcours n’a pas été interrompu ou détourné pour un motif personnel ou indépendant de l’emploi et pendant les voyages ou les déplacements pris en charge par l’employeur [11]. Les statistiques disponibles à la Caisse de Sécurité Sociale (CSS) sénégalaise continuent d’indiquer des proportions élevées de l’ordre de 2.251 AT en 2013 et leur coût représente 4% du PIB tous les ans [12]. Au Sénégal, les travaux de recherche sur les AT voire sur la santé et sécurité au travail n’ont naturellement porté que sur les travailleurs, victimes directs des risques en milieu de travail. Cependant, une maladie grave, une blessure ou la mort liée au travail a aussi des effets psychologiques, sociaux et économiques sur les familles des victimes et des amis proches [13]. C’est pour cette raison que nous avons réalisé cette présente étude afin de décrire les différentes caractéristiques des AT déclarés auprès de la CSS de Dakar et d’étudier leurs conséquences socio-professionnelles, en particulier le devenir socio-professionnel des victimes.

## Méthodes

Il s’agissait d’une étude rétrospective et transversale sur une période de 5 ans (de 2002 à 2006). Neuf mille trois cent huit AT ont été déclarés durant cette période. Nous avions vérifié l’existence des éléments suivants: le procès-verbal, le volet déclaration d’AT, le volet de soins, les différents certificats médicaux et les décisions technico-administratives. Seuls 133 dossiers étaient inclus dans l’étude. Sur l’ensemble des dossiers retenus, nous avions étudié les paramètres suivants: date de l’accident, date de déclaration, identité de la victime (âge, sexe, profession et qualification professionnelle), identité du déclarant (chef d’entreprise ou son représentant, nature de l’établissement et adresse exacte), renseignements relatifs à l’accident (date, heure, lieu, causes matérielles, fait accidentel), les caractéristiques des lésions (type, nature et siège). Les victimes dont les dossiers étaient inclus dans notre étude ont été convoquées puis interrogées sur leur vécus socio-professionnels pré et post accident et les mesures médico-administratives adoptées. Comme paramètres, nous avions retenu: l’âge, le sexe, la profession, la qualification professionnelle, le secteur d’activité, la notion d’accident de trajet, le lieu de travail, la nature et le siège des lésions occasionnées, l’incapacité temporaire de travail, les séquelles, l’incapacité fonctionnelle, la réduction de l’autonomie, le préjudice esthétique, l’invalidité et les conséquences socio-professionnelles.

Les limites de notre travail étaient: 1) Le statut professionnel des victimes est souvent confondu avec la qualification professionnelle et la profession; 2) Il y’a parfois un écart entre le métier et la tache précisément accomplie au moment de l’accident; 3) L’importance des répercussions socio-professionnelles observées et la rareté des données publiées à ce sujet. L’exploitation statistique des données recueillies était effectuée grâce au logiciel SPSS.

## Résultats

### Aspects socio-professionnels

Notre série était composée d’une femme et de 132 hommes. La moyenne d’âge était de 37,55 ans avec des extrêmes 13 et 57 ans. Les travailleurs âgés de moins de 40 ans étaient les plus touchés avec 60,9% (n = 81) des cas (Tableau 1). Selon le poste de travail, 12,8% (n = 17) étaient des machinistes, les matelots représentaient 9,8% des cas (n = 13) et les mécaniciens 7,5% des cas (n = 10). Le BTP était le secteur le plus pourvoyeur d’AT avec 22,6% des cas (n = 28) suivi de la pèche avec 15% des cas (n = 19). Le Tableau 1représente la répartition des AT selon le secteur d’activité. Dans notre série, les AT survenaient le plus souvent au lieu du travail soit dans 77,4% des cas (n = 103) et sur le trajet dans 22,6% des cas (n = 30).

**Tableau 1:** Caractéristiques socio-professionnelles des accidentés

	Fréquence	Pourcentage (%)
**Age**		
Moins de 25 ans	14	10,5
26-30	24	18
31-35	26	19,5
36-40	17	12,9
41-45	18	13,5
46-50	13	9,8
Plus de 50	21	15,8
**Répartition des AT selon le secteur d’activités**		
BTP	28	22,6
Commerce	10	7,7
Concessionnaire automobile	2	1
Hôtellerie/restauration	4	3,1
Industrie alimentaire	12	9,8
Industrie chimique	4	3,1
Industrielles/commerciales	4	3
Manutention	8	6,2
Marine/pêche	19	15
Métallurgie	4	3,1
Mines/énergie	11	8,3
Plastique/caoutchouc/	4	3
Parfumerie/cosmétique	3	2,3
Service public	6	4,6
Textile	4	3,1
Transit	2	0,8
Transport terrestre	4	3,1
Artisanat	4	3
**Répartition des AT selon la profession**		
Machinistes	17	12,8
Chauffeurs	8	6
Gardiens	4	3
Chefs de service vente	2	1,5
Aconiers	2	1,5
Matelots	13	9,8
Manœuvres	7	5,3
Electriciens	3	2,3
Peintres	2	1,5
Frigoristes	2	1,5
Mécaniciens	10	7,5
Comptables	5	3,8
Chaudronniers	3	2,3
Soudeurs	2	1,5
Cuisiniers	2	1,5
Dockers	8	6
Menuisiers ébénistes	4	3
Maitres d’hôtel	2	1,5
Topographes	2	1,5
Fabricants de peintures	2	1,5
Autres (Polyvalents)	33	24,7

### Aspects lésionnels

La main était le siège de prédilection des lésions des AT dans notre série avec 30,1% des cas (n = 40) suivie des membres inférieurs (exclus les pieds) avec 18% des cas et le membre supérieur avec 13,5% des cas (Tableau 2). Les fractures et les amputations étaient les lésions les plus fréquentes avec respectivement 51,7 % et 8,3% des cas. Onze brûlures et 9 plaies ont été dénombrées. Dans notre série, 102 des victimes soit 76,7% des cas se plaignaient d’un préjudice esthétique. La souffrance physique était notée chez 74 victimes soit 56,6% des cas. On a noté une incapacité fonctionnelle chez 39,1% des cas et une réduction de l’autonomie chez 26,3% des victimes. L’évolution des lésions a été marquée par 11 cas de rechute soit dans 8,3% des cas, 10 cas d’ostéites, 7 cas d’amputations et 2 cas de paralysie du membre supérieur. D’autres complications ont été notées telles que: ulcère de jambe, nécrose post traumatique, surdité, baisse de la libido, ischémie, rupture de l’urètre et de cataracte traumatique.

**Tableau 2:** Caractéristiques des lésions

	Fréquence	Pourcentage (%)
**Répartition des AT selon le type des lésions**		
Fracture	64	51,7
Brulure	11	8,3
Amputation	42	31,6
Plaie	6	4,5
Contusion	1	0,8
Entorse	1	0,8
Luxation	1	0,8
Présence de corps étranger	1	0,8
Divers	1	0,8
**Répartition des AT selon le siège des lésions**		
Tête (excepté yeux)	1	0,8
Mains	40	30,1
Pieds	40	30,1
Siege interne	12	9
Yeux	5	3,8
Tronc	1	0,8
Membres supérieurs (excepté mains)	18	13,5
Membres inférieurs (excepté pieds)	23	18
Bassins	4	3
Localisations multiples	28	21,1

### Devenir des accidentés

Au plan professionnel, 81 victimes d’AT soit 60,9% avaient repris la même profession dont 97,5% (n = 79) dans la même entreprise et 2 avaient changé d’entreprise. Trente et une victimes (23,3%) ont été licenciées. Douze des accidentés avaient bénéficiés d’un reclassement professionnel soit dans 9% des cas. Nous avons noté 5 cas de réadaptation professionnelle, 2 cas de retraite normale et un cas de retraite anticipée. Une de nos victimes avait migré dans le secteur informel. Sur le plan social, ces accidents avaient engendré en moyenne une perte de 236,7 jours de travail avec des extrêmes allant de 21 à 1.660 jours. Les taux d’IPP variaient entre 12 et 80% avec une moyenne de 29%. Les victimes percevaient des salaires annuels allant de 360.821 à 8.332.377 Francs CFA (12.721,18 Euros). Le salaire moyen était de 888.817,050 Francs CFA (1.356,97 Euros) par an. Le montant annuel moyen de la rente était de 1.640.329 Francs CFA (2.504,31 Euros) avec un salaire moyen de 75.642 Francs CFA (115,48 Euros). Le salaire journalier minimum était estimé à 1.070,48 Francs CFA (1,63 Euros) et le maximum à 7.560 Francs CFA (11,54 Euros). Parmi les victimes, 23,3% (n = 31) étaient licenciées au décours de l’accident. Ce licenciement était motivé dans 80% des cas (n = 25) par une inaptitude médicale. La majorité de nos licenciés (90%) n’avaient pas trouvé un autre emploi, ce qui admettait des répercussions au sein des familles où 72% (n = 22) des conjoints étaient sans activité professionnelle et 59% (n = 18) avait une charge familiale avec en moyenne 4 enfants à charge. Aucune mesure d’accompagnement n’avait été envisagée lors de la mise en place de l’inaptitude.

## Discussion

Comprendre comment les victimes d’AT perçoivent la gravité de leur accident et leur capacité à faire face aux blessures résultant et les conséquences liées à l’emploi sont des prédicteurs pour le retour au travail. La présente étude a étudié les conséquences socio-professionnelles en particulier le devenir socio-professionnel des victimes d’un AT au Sénégal de 2002 à 2006 auprès de la CSS. Notre population d’étude était exclusivement masculine (132 hommes et une femme). Cette surreprésentation masculine peut se justifier par le petit effectif des femmes dans les secteurs professionnel les plus pourvoyeurs d’AT dans notre contexte, le BTP et la pèche. La majorité des accidents survenaient aux postes nécessitant plus d’effort physique (mécanicien, machinistes, matelots, manœuvre) donc réservés aux hommes et que les femmes sont en général exclus des emplois de terrain dans les secteurs à risque. Elles occupent le plus souvent des postes moins à risque tels que: secrétaires, gérantes ou cadres de direction. Nos résultats rejoignent ceux de plusieurs travaux de la littérature [3, 6, 14-19]. Les AT restent le problème majeur de santé et sécurité au travail dans le secteur du BTP, on parle de secteur à plus haut risque [16]. Dans notre série, la pêche représente la deuxième cause d’AT, ceci s’explique en partie par les caractéristiques particulières du milieu de travail: mouvements de navires, intempéries et courants marins, brouillards, collision, engins, incendie… [20]. Les jeunes étaient les plus touchés dans notre série. La sous qualification et le manque d’expérience chez ces jeunes expliquent en partie la grande fréquence des AT d’une part. D’autre part, les plus âgés sont vraisemblablement placés dans des postes de direction et donc, peut-être à un faible risque d'encourir des AT. Tadesse [17] en Ethiopie a trouvé plus d’accidents chez les employés âgés de moins de 35 ans et moins expérimentés. D’autres auteurs ont fait le même constat [14, 18, 19, 21]. Dans cette étude, les machinistes étaient les plus touchés suivis des matelots et mécaniciens. Plusieurs études ont retenu l’utilisation de machine et les taches d’entretien des machines comme cause fréquente des AT [19, 22-24].

Dans notre série, la majorité des AT survenaient au lieu du travail. Ceci s’explique par le fait que les tâches dans les secteurs du BTP et de la pêche, les plus exposant aux AT, n’ont lieu dans la quasi-totalité que sur les chantiers et bateaux de pêche et donc sur les lieux de travail et rarement sur le trajet menant au lieu de travail. Les secteurs les plus concernés dans notre série sollicitent des travaux manuels. Ceci explique l’atteinte fréquente des mains lors des accidents. Le membre inférieur (exclu le pied) occupait le deuxième siège des lésions dans notre étude suivie du membre supérieur (exclu la main). Ces résultats sont comparables à ceux de Tadesse [17]. Les fractures étaient la lésion prédominante dans notre étude en concordance avec plusieurs séries de la littérature [14, 17, 18, 21, 22]. Dans cette étude, la durée moyenne d’arrêt de travail était de 236,7 jours. Nos résultats concordent avec ceux de plusieurs travaux [14, 24, 25]. La reprise de l’activité professionnelle est fréquemment observée dans notre série. Cela rejoint les données de la littérature [14, 26]. Parmi les facteurs influençant la durée de l’invalidité et le retour au travail, nous citons: les caractéristiques personnelles de la victime et de sa famille, les caractéristiques de l'emploi et la mesure dans laquelle la rémunération est reçue. Tous ces facteurs sont déjà décrits par certains auteurs [27, 28]. Dans notre série, le licenciement était motivé par l’inaptitude médicale. Une grande partie de ces licenciés était restée par la suite sans emploi, ceci illustre les coûts indirects des accidents du travail: perte d’emploi, perte de revenu, dislocation du tissu familial [9, 10]. L’absence de mesure d’accompagnement et de programme d’aide rend les victimes vulnérables. Les AT ont beaucoup de conséquences, y compris le paiement de la rémunération des prestations des travailleurs, les coûts économiques pour l'employeur, la prestation des services de soins médicaux et l'incapacité de travailler pour le travailleur. Des études ont démontré que les AT ont des effets sur le bien-être et le comportement psychologique des victimes [27-30].

## Conclusion

Notre étude montre l’ampleur des AT dans nos entreprises, ce qui nous pousse à mettre sur place des programmes d’intervention ayant pour but de réduire les incapacités fonctionnelles et les souffrances des victimes. Ainsi, l’employeur doit être le premier acteur de la prévention en veillant sur les principes, généraux de prévention définis par la réglementation. Des politiques cohérentes d’aide et de soutien devront être mises en place pour un accompagnement social des victimes et de leurs familles.

### Etat des connaissances actuelles sur le sujet

- La déclaration des accidents du travail au niveau de la Caisse de Sécurité Sociale sénégalaise est une obligation légale pour toutes les entreprises exerçant dans le pays;
- Plusieurs études ont été réalisées sur les accidents du travail au Sénégal et n’ont porté que sur les caractéristiques socio-professionnelles, cliniques et thérapeutiques;
- Dans le monde, plusieurs auteurs ont démontré que les accidents du travail ont des effets sur le bien-être et le comportement psychologique des victimes ainsi que leur famille.

### Contribution de notre étude à la connaissance

- Cette étude s’intéresse particulièrement sur les déterminants liés à l’accident, les caractéristiques des lésions et l’impact des accidents sur la carrière professionnelle des victimes;
- Il s’agit aussi d’apprécier le coût indirect de ces accidents au niveau de la vie familiale des accidentés;
- Notre étude a apprécié les effets psychologiques, sociaux et économiques sur les familles des victimes d’AT dans notre pays.

## Conflits d’intérêts

Les auteurs ne déclarent aucun conflit d’intérêts.

## References

[1] Karvonen M, Karvonen M, Mikheev MI (1986). Epidemiology in the context of occupational health. Epidemiology of Occupational Health. WHO.

[2] Hamalainen P (2009). The effect of globalization on occupational accidents. Saf Sci.

[3] Larsson TJ, Field B (2002). The distribution of occupational injury risks in the Victorian construction industry. Saf Sci.

[4] Lowery JT, Glazner J, Borgerding JA, Bondy J, Lezotte DC, Kreiss K (2000). Analysis of construction injury burden by type of work. Am J Ind Med.

[5] Bureau international du travail (BIT) Rapport IV, Cadre promotionnel pour la sécurité et la santé au travail, 93ème session 200 Brisson.

[6] Gingras S, Vezina M, Bernard PM (1992). Les accidents du travail sur les chantiers de construction de la baie James entre 1976 et 1986, Rapport de recherche.

[7] Loewenson R (2001). Mondialisation et santé au travail: l’exemple de l’Afrique australe. Bull OMS.

[8] Robert R (2000). Les accidents du travail: guide pratique médico administratif et juridique, collection Minidroit.

[9] Leburn T, Sailly JC (1992). L’évaluation médico-économique des stratégies diagnostiques et thérapeutiques. Problèmes Economiques.

[10] Drunimond MF, Stoddard GL, Torrance GW (1987). Methods for economic evaluation of health Care programmes.

[11] République du Sénégal Code de la Sécurité Sociale.

[12] Fédior O (2015). Accidents de travail: un trou de 4% dans le PIB.

[13] Matthews LR, Bohle P, Quinlan M, Rawlings-Way O (2012). Traumatic death at work: consequences for surviving families. Int J Health Serv.

[14] Maoua M, Aroui H, Boughattas W, Brahem A, Guedri S, Kalboussi H (2016). Accident du travail avec traumatisme grave du coude: devenir professionnel des victimes. Arch Mal Prof Env.

[15] Laflamme L, Eilert-Petersson E (2001). Injury risks and socioeconomic groups in different setting, differences in morbidity between men and women at working ages. Eur J Public Health.

[16] Marouen-Jamoussi S, Loukil-Feki M, Masmoudi A, Kammoun L, Zouari C, Jmal-Hammami K (2006). Les accidents du travail mortels dans le secteur privé en Tunisie. Arch Mal Prof Env.

[17] Tadesse S, Israel D (2016). Occupational injuries among building construction workers in Addis Ababa, Ethiopia. J Occup Med Toxicol.

[18] Abas AB, Mohd Said DA, Aziz Mohammed MA, Sathiakumar N (2013). Fatal occupational injuries among non-governmental employees in Malaysia. Am J Ind Med.

[19] Abas AB, Said AR, Mohammed MA, Sathiakumar N (2011). Non-fatal occupational injuries among non-governmental employees in Malaysia. Int J Occup Environ Health.

[20] International Labour Organization (ILO) (1999). Safety and health in the fishing industry Report for discussion at the Tripartite Meeting on Safety and Health in the Fishing Industry.

[21] Husberg BJ, Fosbroke DE, Conway GA, Mode NA (2005). Hospitalized nonfatal injuries in the Alaskan construction industry. Am J Ind Med.

[22] Solomon C (2002). Accidental injuries in agriculture in the UK. Occup Med.

[23] Pickett W, Brison RJ, Niezogoda H, Chipman ML (1995). Non-fatal farm injuries in Ontario: a population based survey. Accid Anal Prev.

[24] Bouhlel M, Brahem A, Khalfaoui D, Boughattas W, Maoua M, Rejeb K (2016). Les accidents de travail lombaires: réparation et devenir professionnel. Arch Mal Prof Env.

[25] Bureau of Labor Statistics (2003). United States Department of Labor. Table 2: Numbers of nonfatal occupational injuries and illnesses by industry and case types, 2002.

[26] Datié AM, Nandjui MB (2012). Réadaptation médicale en Côte d’Ivoire: acquis, défis et perspectives, 50 ans après les indépendances. J Readapt Med.

[27] Dembe AE (2001). The social consequences of occupational injuries and illnesses. Am J Ind Med.

[28] MacKenzie EJ, Morris JA, Jurkovich GL, Yasui Y, Cushing BM, Burgess AR (1998). Return to work following injury: the role of economic, social and jobrelated factors. Am J Public Health.

[29] Keogh J, Nuwayhid I, Gordon J, Gucer P (2000). The impact of occupational injury on injured worker and family: outcomes of upper extremity cumulative trauma disorders in Maryland workers. Am J Ind Med.

[30] Pransky G, Benjamin K, Hill-Fatouhi C, Himmelstein J, Fletcher K, Katz J, Johnson W (2000). Outcomes in work-related upper extremity and low back injuries: results of a retrospective study. Am J Ind Med.

